# Methylmercury Epigenetics

**DOI:** 10.3390/toxics7040056

**Published:** 2019-11-09

**Authors:** Megan Culbreth, Michael Aschner

**Affiliations:** 1 Department of Environmental Medicine, University of Rochester, Rochester, NY 14642, USA; 2 Department of Molecular Pharmacology, Albert Einstein College of Medicine, 1300 Morris Park Avenue, Bronx, NY 10461, USA

**Keywords:** methylmercury (MeHg), epigenetics, microRNA (miRNA), histone modifications, DNA methylation, transgenerational inheritance

## Abstract

Methylmercury (MeHg) has conventionally been investigated for effects on nervous system development. As such, epigenetic modifications have become an attractive mechanistic target, and research on MeHg and epigenetics has rapidly expanded in the past decade. Although, these inquiries are a recent advance in the field, much has been learned in regards to MeHg-induced epigenetic modifications, particularly in the brain. In vitro and in vivo controlled exposure studies illustrate that MeHg effects microRNA (miRNA) expression, histone modifications, and DNA methylation both globally and at individual genes. Moreover, some effects are transgenerationally inherited, as organisms not directly exposed to MeHg exhibited biological and behavioral alterations. miRNA expression generally appears to be downregulated consequent to exposure. Further, global histone acetylation also seems to be reduced, persist at distinct gene promoters, and is contemporaneous with enhanced histone methylation. Moreover, global DNA methylation appears to decrease in brain-derived tissues, but not in the liver; however, selected individual genes in the brain are hypermethylated. Human epidemiological studies have also identified hypo- or hypermethylated individual genes, which correlated with MeHg exposure in distinct populations. Intriguingly, several observed epigenetic modifications can be correlated with known mechanisms of MeHg toxicity. Despite this knowledge, however, the functional consequences of these modifications are not entirely evident. Additional research will be necessary to fully comprehend MeHg-induced epigenetic modifications and the impact on the toxic response.

## 1. Introduction

In recent years, there has been a surge in evidence that heavy metals can disrupt characteristic epigenetic programming in order to elicit toxicity [1]. Methylmercury (MeHg) potentially is one such heavy metal. A pervasive environmental toxicant, MeHg is well-established to target the developing nervous system [2]. Given that MeHg exerts toxicity during development, it seems plausible epigenetics might play a role, as epigenetic modifications underline several developmental processes [3]. As such, researchers have started to investigate MeHg-stimulated epigenetic modifications. Thus far, however, there has yet to be a comprehensive review of these studies.

Epigenetic modifications cause heritable changes in gene expression without mutating the DNA sequence [4]. Possible modifications include variation in microRNA (miRNA) expression, histone acetylation or methylation, and DNA hypo- or hypermethylation. miRNAs are small, non-coding RNA molecules, which control gene expression post-transcriptionally [5]. Acetylation or methylation of lysine residues in histone 3 and histone 4 of the nucleosome are the most common known histone modifications, and generally prompt an active or repressed chromatin state, respectively [6]. DNA methylation catalyzed by DNA methyltransferases appends a methyl group to cytosine nucleotides, typically within promoter sequences, and often is associated with gene silencing [7].

There is a well-defined epigenetic association between prenatal environmental exposures and adverse neurodevelopmental outcomes [8]. As MeHg can cross the placental barrier [9], characteristic neurobehavioral deficits observed resultant to exposure [10,11,12] might have epigenetic foundations. Moreover, exposure not only occurs to the mother and child, but also to the next generation germline. Thus, transgenerational inheritance of MeHg-induced epigenetic modification is conceivable. Humans are primarily exposed to MeHg through consumption of contaminated seafood [13]. Consequently, the potential for MeHg-induced epigenetic modifications within human populations is vast.

For the purposes of this review, only controlled in vitro and in vivo laboratory studies were included. In this manner, potential confounders of environmental exposures could be eliminated, and effects solely attributed to MeHg exposure. However, human epidemiological studies are discussed as a point of comparison. In these reports, potential effects of other environmental contaminants cannot be discredited. Thus far, MeHg epigenetic studies have predominately focused on neurodevelopment, however, some other tissues have been examined. miRNA expression, histone modifications, and DNA methylation have all been observed to be altered consequent to exposure. Although, some effects can be associated with known mechanisms of MeHg toxicity, the functional consequence of these epigenetic modifications are not entirely evident.

## 2. Epigenetic Modifications In Vitro

Table 1 summarizes the MeHg-induced epigenetic modifications that have been identified in vitro. Thus far, these studies have been limited to neural derived cells. Regardless, effects on miRNA expression, histone modifications, and DNA methylation were observed. The concentration and duration of MeHg exposure varied between studies; however, potentially convergent effects could be ascertained. Furthermore, the in vitro data summarized here, support existing knowledge regarding the mechanism of MeHg toxicity.

Human stem cell-derived neurons and glia exposed to MeHg during neuronal differentiation displayed increased miR-302b, miR-367, miR-372, miR-141, and miR-196b expression [14]. miR-302b, miR-367, and miR-372 expression have been associated with a stem cell pluripotent phenotype [15], while miR-141 was involved in cell differentiation [16]. miR-196b downregulates *hox* gene translation [17]. Intriguingly, exposed human neural progenitor cells exhibited decreased miR-1285, miR-25, and miR-30d expression [18]. These miRNAs target the tumor suppressor p53 [19,20,21]. MeHg has been recognized to inhibit neuronal cell proliferation and differentiation [22]. The effects on miRNA expression outlined here, support the hypothesis that MeHg indirectly alters neurodevelopment through epigenetic modifications.

Experiments in human-derived SH-SY5Y neuroblastoma cells and rat cortical neurons detected increased histone deacetylase 4 (HDAC4) protein resultant to MeHg exposure [23]. Moreover, this correlated with increased HDAC4 binding to the brain-derived neurotrophic factor (BDNF) promoter [23] and decreased global histone 4 (H4) acetylation in SH-SY5Y cells [24]. Further, a concurrent decrease in miR-206 expression was revealed in rat cortical neurons [25]. miR-206 was previously found to downregulate HDAC4 [26] and BDNF [27] protein. MeHg has been established to decrease BDNF gene expression concomitant with neurobehavioral deficits [28]. As such, decreased BDNF promoter acetylation resultant to enhanced histone deacetylase activity, potentially underscores this toxic response.

Tyrosine hydroxylase (TH) was also found to be altered in human fetal brain-derived cells exposed to MeHg during neuronal differentiation. These cells exhibited increased histone 3 (H3) lysine 27 (K27) trimethylation, at the TH promoter [29]. H3K27 trimethylation is a repressive histone mark associated with reduced gene transcription [30]. TH overexpression in SH-SY5Y cells was previously found to attenuate MeHg-induced cytotoxicity [31]. Further, perinatal MeHg exposure in vivo did not alter TH gene expression [32]. Thus, repression of TH gene transcription might be one possible mechanism of MeHg-induced neurotoxicity.

Finally, rat cortical neural stem cells exposed to MeHg displayed decreased global DNA methylation, which correlated with reduced DNA methyltransferase (DNMT)-3b gene expression [33]. DNA methylation typically associates with gene repression, although not exclusively [34], while DNMT-3b de novo methylates DNA, particularly during embryonic development [35]. Therefore, MeHg exposure has potential to upregulate aberrant gene expression broadly, at least in brain-derived cells, which could result in undetermined functional effects. Moreover, the possible impact enhanced global gene expression might have on neurodevelopment is immense [36].

## 3. Epigenetic Modifications In Vivo

Table 2 summarizes the MeHg-induced epigenetic modifications that have been identified in vivo. These studies employed one invertebrate model, *Caenorhabditis elegans* (*C. elegans*), and several diverse vertebrate models, which included zebrafish, mice, rats, and mink. Multiple MeHg exposure routes at different developmental time-points were assessed, and unlike the in vitro studies, multiple organs (brain, liver, kidneys) were examined. Intriguingly, continuity between the in vitro and in vivo studies can be perceived. Furthermore, these in vivo reports also reinforce known mechanisms of MeHg toxicity.

As mentioned in the previous section, global H4 acetylation was reduced in SH-SY5Y cells. In the same study, adult male C57Bl/6 mice also exhibited decreased H4 acetylation in the cerebellum and cortex [24]. Moreover, perinatally exposed C57Bl/6 mice displayed increased H3K27 trimethylation and decreased histone 3 (H3) acetylation at the BDNF promoter. This further correlated with hypermethylation of the BDNF promoter in the hippocampus [28]. MeHg appears to affect histone acetylation both globally and at the individual gene level in the brain. Likewise, a specific effect on BDNF promoter methylation, could be extended, as exposed juvenile male mink presented with decreased global DNA methylation and reduced DNMT activity in the cortex [37]. These results emphasize MeHg potentially indiscriminately modifies the neural epigenetic profile, irrespective of developmental time-point. Exposure paradigm inconsistencies between studies, however, confound comparisons.

miRNA-seq analysis on small RNAs isolated from *C. elegans* exposed to MeHg from embryos to larval stage 4 (L4) revealed decreased miR-37-3p, miR-41-5p, miR-70-3p, and miR-75-3p expression; however, putative miRNA targets were unable to be identified [38]. Despite target uncertainty, aberrant miRNA expression can result in dysregulation of post-transcriptional gene expression [39]. Interestingly, zebrafish embryos exposed 48 hours post-fertilization (hpf) for 24 hours (h) to MeHg also exhibited variable miRNA expression; dre-miR-7147 and dre-miR-26a expression were decreased, while dre-miR-375 and dre-miR-206 expression were increased [40]. Hu and colleagues did perform miRNA-pathway-network analysis to identify potential targets; however, a combined MeHg and silica nanoparticle exposure was assessed, and not MeHg alone [40]. Regardless, it is difficult to determine tissue-specific effects from the above mentioned studies, as RNA was isolated from whole larvae [38] and embryos [40]. Therefore, the increased dre-miR-206 observed in zebrafish embryos [38] versus the decreased miR-206 in rat cortical neurons [25], does not necessarily reflect a real model system difference.

Increased histone 3 (H3) lysine 4 (K4) trimethylation (H3K4me3) was observed in *C. elegans* larvae exposed to MeHg from L1 to L4 [41]. H3K4me3 typically is associated with actively transcribed genes [42]. Contrary to predicted global gene repression in the brain [24,37], these results possibly indicate MeHg exposure increases gene expression overall in the whole organism. Furthermore, Rudgalvyte and colleagues found enhanced H3K4me3 in glutathione S-transferase (gst) genes [41]. Glutathione S-transferase catalyzes glutathione conjugation reactions, a major detoxification mechanism [43]. MeHg has been demonstrated to enhance glutathione S-transferase activity in the liver. Interestingly, this was concurrent with reduction in the required substrate glutathione [44]. Potentially, a threshold at which glutathione depletion supersedes enhanced enzymatic activity resultant to MeHg exposure exists. The global implications of this toxic response, however, remain to be explored.

Finally, Sprague-Dawley rats exposed to MeHg from gestation day 1 (GD 1) to post-natal day 21 (PND 21) [45], as well as adult female zebrafish exposed in the diet [46], both exhibited no effect on global DNA methylation in the liver. Interestingly, however, DNMT-1 and DNMT-3b gene expression were both reduced in the former report [45]. Possibly MeHg exposure impacts individual gene methylation patterns uniquely in the liver, but not globally. Exon 1 of matrix metalloproteinase 9 (MMP9) was found to be hypomethylated in adult female Wister rats, albeit in the kidneys [47]. Thus, MeHg exposure potentially imparts epigenetic modifications at single genes at least in the liver and kidneys.

## 4. Transgenerational Inheritance

Table 3 summarizes MeHg effects on transgenerational inheritance. Thus far, there have been three studies that examined MeHg effects on transgenerational inheritance in zebrafish; two of the these reports involved exposure directly to the embryo (F1) [48,49], while the other was to adult females (F0) fed a MeHg-enriched diet [46]. However, only Xu and colleagues assessed MeHg-induced modifications in the F3 generation [49], the first generation not directly exposed [50]. As such, effects observed in Carvan et al., 2017 [48] and Olsvik et al., 2014 [46] are not necessarily transgenerationally inherited, as direct exposure to the F1 embryo and F2 germline occurred.

Like the adult female zebrafish, the F1 and F2 offspring did not exhibit effects on global DNA methylation in the liver. However, some single genes were found to be hypo- or hypermethylated in the F1 and F2 generations. Intriguingly, however, selenoprotein P (SEPP1) expression was unaltered in any generation [46]. SEPP1 aids in selenium homeostasis [51]. MeHg was previously observed to decrease selenium transport in zebrafish larvae [52]. Moreover, SEPP1 was identified as a major serum mercury (Hg) transporter in MeHg-intoxicated rats [53]. Undoubtedly, it is possible effects on SEPP1 might not be transgenerational inherited; however, an absence of altered expression in the F0 generation would be unexpected. It has yet to be determined whether the time and duration of exposure contribute to this differential result.

Although, Carvan and colleagues did not extend analysis to the F3 generation, increased sperm epimutations were detected in the F2 generation [48]. As such, epimutations in the F2 germline, hypothetically, would be expected to be inherited by the F3 generation. Xu and colleagues detected no difference in avoidance response or crossing latency in the F2 and F3 generations [49]. These behaviors are indicators of impaired learning [54]. MeHg exposure has previously been demonstrated to induce learning deficits [55]. Thus, neurobehavioral effects observed in one generation have potential to persist in subsequent generations, not necessarily directly exposed to MeHg.

To our knowledge, there have yet to be any mammalian studies that exclusively investigate MeHg transgenerational inheritance. There has, however, been one study that examined MeHg and cadmium co-exposure in mice. Although, MeHg-specific effects cannot be delineated, this co-exposure resulted in impaired glucose tolerance, particularly in matrilineally descended males [56]. Interestingly, minimal effects were observed in the females [56], possibly highlighting sex-specific differences. MeHg has previously been observed to differentially affect males and females [57]. Lack of MeHg-only exposure data, however, makes it difficult to determine whether sex-specific effects are transgenerationally inherited.

## 5. DNA Methylation in Human Populations

Table 4 summarizes MeHg effects on DNA methylation in human populations. These studies primarily associated exposure with single gene hypo- or hypermethylation. Exposure biomarkers in mother-infant pair populations included maternal hair and toenail, as well as cord blood. Cord blood has previously been demonstrated to be a more accurate measure of prenatal MeHg exposure than maternal hair [58]. For adult populations, hair and urine were the exposure biomarkers. Only one study population (Faroe Islands Cohort) was outside the United States.

Some observed effects did contrast with the in vivo data. Those studies [59,60], however, were in adult populations, not in developmentally exposed groups like the in vivo reports. Hair Hg concentration was associated with SEPP1 hypomethylation in male dental professionals [59]. Not only might this underscore potential sex-specific effects, but also may emphasize a sensitive exposure window, as SEPP1 was unaltered in *in ovo* exposed zebrafish [46]. Hair and urine Hg concentration was also associated with glutathione S-transferase (GSTM 1/5) promoter hypermethylation in women undergoing in vitro fertilization (IVF) [60]. In contrast, enhanced H3K4me3 was observed in exposed *C. elegans* larvae [41]. This differential result possibly indicates an exposure threshold at which MeHg-induces glutathione S-transferase activity. Furthermore, glutathione S-transferase might not be as active in adults relative to developing organisms.

Cord blood biomarker was not associated with CpG island methylation changes; however, maternal hair was associated with five CpG site methylation changes in the Faroese birth cohort [61]. CpG islands are typically located in gene promoters, and site methylation represses gene expression [62]. Intriguingly, another maternal biomarker, toenail, was also associated with CpG site methylation changes; the north shore regions of CpG islands were hypermethylated [63]. Shore regions flank CpG islands [64]. Possibly maternal exposure biomarkers more readily associate with general CpG site changes relative to specific genes. Interestingly, an overlapping differentially methylated region (DMR) in TCEANC2 (transcription elongation factor A N-terminal and central domain containing 2) was associated with cord blood biomarker [65]. Moreover, paraoxonase 1 (PON1) hypomethylation was also associated with this exposure biomarker specifically in males [66]. As cord blood is a more robust measure of prenatal exposure, potentially these single gene changes more accurately reflect MeHg effects. Of note, infant toenail, unlike maternal toenail, was associated with a specific gene, EMID2 (collagen type XXVI alpha 1 chain) hypomethylation [67]. This further indicates maternal biomarkers possibly associate with non-specific MeHg-induced epigenetic modifications. 

## 6. Conclusions

Based on the current literature, it would be difficult to make a general conclusion about MeHg-induced epigenetic modifications. Dependent on the tissue or species examined, differential effects were observed, and not necessarily consistent across model systems. Time and route of exposure also impacted results. Thus, these factors must be considered in comparisons with previous and future research. Some associations with known mechanisms of MeHg toxicity can be made but are still limited without further investigation. Despite knowledge gaps, MeHg undoubtedly induces epigenetic modifications, and these modifications have potential to affect resultant toxicity.

In the brain, MeHg seems to induce epigenetic modifications, which disrupt typical neuronal differentiation. These results correlate well with known effects on this developmental process [22]. Interestingly, global DNA methylation decreases; however, overall gene repression appears to occur. Furthermore, transgenerational inheritance of neurobehavioral deficits seems plausible. There is not enough data currently, however, to draw conclusions about effects in other tissues. Moreover, it would be difficult to determine a mechanistic foundation from whole organism investigations. Although, inconsistencies between in vitro data and human studies could be discerned, at this point there is limited evidence to validate concerns for species extrapolation.

## Figures and Tables

**Table 1:** Methylmercury-induced epigenetic modifications in vitro.

Epigenetic Modification	Model	Effect	Dose and Duration	Reference
**miRNA**	rat cortical neurons	decreased miR-206 expression	1 μM MeHg for 12 or 24 h	[25]
	carcinoma, pluripotent human stem cell-derived neurons and glial cells	increased miR-302b, miR-367, miR-372, miR-196b, and miR-141 expression	400 nM MeHg during neuronal differentiation (day 2 to 36 in vitro)	[14]
	immortalized human embryonic neural progenitor cells	decreased miR-1285, miR-25, and miR-30d expression	50 nM MeHg for 24 h	[18]
**Histone modifications**	Human fetal brain-derived immortalized cells	increased H3K27 trimethylation at TH promotor	1 nM MeHg during neuronal differentiation (day 2 to 8 in vitro)	[29]
	SH-SY5Y human neuroblastoma cells	decreased global H4 acetylation	1 μM MeHg for 24 h	[24]
	SH-SY5Y human neuroblastoma cells	increased HDAC4 mRNA, protein, and binding to BDNF promotor	1 μM MeHg for 24 h	[23]
	rat cortical neurons	increased HDAC4 protein	1 μM MeHg for 24 h	
**DNA methylation**	rat cortical neural stem cells	decreased global DNA methylation; decreased DNMT-3b mRNA	2.5 or 5 nM MeHg for 48 h	[33]

**Table 2:** Methylmercury-induced epigenetics modifications in vivo.

Epigenetic Modifications	Model	Effect	Dose and Duration	Reference
**miRNA**	zebrafish	decreased dre-miR-7147 and dre-miR-26a; increased dre-miR-375 and dre-miR-206 expression	microinjected 48 hpf embryos with 0.01 mg/ml MeHg for 24 h	[40]
	*Caenorhabditis elegans*	decreased miR-37-3p, miR-41-5p, miR-70-3p, and miR-75-3p expression	10 μM MeHg from embryo to L4 stage	[38]
**Histone modifications**	*Caenorhabditis elegans*	increased H3K4 trimethylation	10 μM MeHg to L1 to L4 stage	[41]
	adult male C57Bl/6 mice (neural)	decreased H4 acetylation	subcutaneous injection of 10 mg/kg MeHg for 10 days	[24]
	C57Bl/6 mice (neural)	increased H3K27 trimethylation and decreased H3 acetylation at BDNF promotor	dams exposed to 0.5 mg/kg MeHg in drinking water from GD 7 to PND 7	[28]
		hypermethylation of BDNF promotor		
**DNA methylation**	juvenile male mink (neural)	decreased global DNA methylation	0.1–2 mg/kg in diet for 3 months	[37]
		decreased DNMT activity	0.5–2 mg/kg in diet for 3 months	
	Sprague-Dawley rats (hepatic)	decreased DNMT-1 and DNMT-3b mRNA; no effect on global DNA methylation	dams exposed from GD 1 to PND 21 in diet to 2 mg/kg MeHg	[45]
	adult female Wister rats (nephro)	hypomethylation of exon 1 of MMP9; increased MMP9 mRNA and protein	0.5 or 5 ppm MeHg for 28 days by oral gavage	[47]
	zebrafish (hepatic)	no effect on global DNA methylation	adult females fed 10 mg/kg in diet for 47 days	[46]

**Table 3:** Methylmercury effects on transgenerational inheritance.

Model	Effect	Dose and Duration	Reference
CD-1 mice	matrilineally descended F2 and F4 males had elevated blood glucose matrilineally descended F2, F3, and F4 males had higher abdominal adipose tissue weights; increased IRS1 phosphorylation at Ser307 matrilineally and patrilineally F2 descended females had increased kidney weight	adult female mice subcutaneously injected with combination cadmium and MeHg (2 mg/kg) from 4 days before to 4 days after conception	[56]
zebrafish	visual deficits and hyperactivity in F2 increased potassium current amplitude in F2 increased sperm epimutations (30 nM) in F2	embryos exposed to 0, 1, 3, 10, 30, or 100 nM MeHg until 24 hpf	[48]
zebrafish	no difference in avoidance response or crossing latency at 0.1 μM from controls in F2 and F3 no difference in avoidance response or crossing latency at 0.01 μM from controls in F2	embryos exposed to 0, 0.01, 0.10 μM MeHg from 2 to 24 hpf	[49]
zebrafish	no effect on global DNA methylation in F0, F1, or F2 liver some genes hypo- or hypermethylated in F1, and 1 gene hypermethylated in F2 no effect on SEPP1 expression across F0–F2	adult females fed 10 mg/kg MeHg in diet for 47 days	[46]

**Table 4:** Methylmercury effects on DNA methylation in human populations.

Population	MeHg Measurement	Effect	Reference
newborns (Baltimore, MD, USA)	cord blood	MeHg concentration associated with overlapping DMR within TCEANC2	[65]
mother-infant pairs (USA)	maternal toenail	associated with hypermethylated north shore regions of CpG islands	[63]
mother-child pairs (Massachusetts, USA)	maternal blood	associated with lower regional cord blood DNA methylation at PON1 in males at 2.9–4.9 years	[66]
dental professionals (Michigan, USA)	hair and urine	hair Hg associated with *SEPP1* hypomethylation in males	[59]
women undergoing IVF (San Francisco, CA, USA)	hair and urine	associated with *GSTM 1/5* promotor hypermethylation	[60]
Faroese birth cohort	cord blood and maternal hair	no CpG site methylation changes associated with cord blood; 5 CpG site methylation changes associated with maternal hair	[61]
infants (Rhode Island, USA)	infant toenail	associated with *EMID2* hypomethylation in infant placenta	[67]
